# Role of Noncoding RNA in Development of Nonalcoholic Fatty Liver Disease

**DOI:** 10.1155/2019/8690592

**Published:** 2019-02-26

**Authors:** Ruixian Huang, Xiaoyan Duan, Jangao Fan, Guangming Li, Baocan Wang

**Affiliations:** Center for Fatty Liver, Department of Gastroenterology, Xin Hua Hospital affiliated to Shanghai Jiao Tong University School of Medicine, Shanghai 200092, China

## Abstract

Nonalcoholic fatty liver disease (NAFLD) is increasing in prevalence globally, but little is known about its specific molecular mechanisms. During the past decade, noncoding RNAs (ncRNAs) have been linked to NAFLD initiation and progression. They are a class of RNAs that play an important role in regulating gene expression despite not encoding proteins. This review summarizes recent research on the relationship between ncRNAs and NAFLD. We discussed the potential applicability of ncRNAs as a biomarker for early NAFLD diagnosis and assessment of disease severity. With further study, ncRNAs should prove to be valuable new targets for NAFLD treatment and benefit the development of noninvasive diagnostic methods.

## 1. Introduction

Nonalcoholic fatty liver disease (NAFLD) is the most common chronic liver disease in developed countries due to nonalcohol consumption of liver lipids. The disease exhibits a characteristic spectrum of liver damage, ranging from simple steatosis to nonalcoholic steatohepatitis (NASH), liver fibrosis, and possibly cirrhosis or even hepatocellular carcinoma (HCC) [1]. Multiple factors, including heredity, eating habits, lifestyle, and metabolic changes, all interact to induce hepatocyte injury and liver disease progression. Despite considerable research, NAFLD pathogenesis has remained unclear. However, data from the past decade have revealed that noncoding RNAs (ncRNA) may play important regulatory roles in NAFLD initiation and progression. This class of molecules, including microRNA (miRNA), long noncoding RNA (lncRNA), and circular RNA (circRNA), does not encode proteins but still influences gene expression [2].

MicroRNAs regulate posttranscriptional gene expression and are important in adipocyte differentiation, lipid metabolism, cholesterol metabolism, insulin resistance, and immune response. In vitro and in vivo models of NAFLD have shown that miRNAs affect the regulation of fatty acids (FA) and cholesterol metabolism in the liver. In addition, they are involved in the process of regulating oxidative stress, inflammation, and apoptosis [3, 4].

Long noncoding RNAs are over 200 nucleotides. They appear to function as transcriptional regulators of gene activation or silencing through chromatin modification [5]. Posttranscriptional regulation through lncRNA involves base pairing with mRNA and using RNA-binding protein/miRNA as bait to inhibit splicing [6]. The pathophysiology of numerous diseases and cancers has been linked to lncRNA [7], but the connection with NAFLD, as well as other metabolic syndromes (e.g., obesity, insulin resistance, type 2 diabetes), remains relatively unexplored [8]. We are only beginning to understand the role of lncRNAs in NAFLD steatosis and fibrosis.

Finally, circRNA is a covalently closed-loop structure, unlike typical linear RNA that terminates with a 5′ cap and a 3′ tail. Circular RNAs are considered the main subunit of gene transcription [9]. Unsurprisingly, they are abundant, stable, and widely expressed in mammalian cells. Additionally, circRNAs exhibit cell type specificity, tissue specificity, and specific expression at different developmental stages. Recent studies have also linked circRNAs to NAFLD pathophysiology.

In light of recent scientific developments, this review aims to summarize and elucidate the involvement of noncoding RNA in NAFLD.

## 2. MiRNAs

First described in* Caenorhabditis elegans*, miRNAs are endogenous, small noncoding RNAs of approximately 18–25 nucleotides [10], with a strictly controlled, multistep biosynthesis [11]. Initial miRNAs are transcribed into primary miRNA (pri-miRNA) via the action of RNA polymerase Pol II [12]. These pri-miRNAs are then processed into pre-miRNAs, hairpin structures formed via DROSHA and DICER, two ribonucleases. DROSHA cuts into the nucleus to produce pre-miRNA, which is transported from the export protein-5 (Exp5)/Ran-GTP complex to the cytoplasm. Here, DICER-TRBP-PACT cleaves pre-miRNA to produce a mature miRNA comprising a guide strand and passenger chain (or miRNA *∗*) [13, 14]. The guide chain is loaded onto Argonaute 2 (AGO2) and miRNA *∗* is generally considered to be degraded. Mature miRNA, AGO2, and GW182 are then loaded onto RNA-induced silencing complexes (RISC), where they bind to target mRNA [11]. This action then inhibits protein synthesis, while also adenylating and degrading the bound mRNA [15, 16]. However, although miRNA *∗* degradation is generally thought to be the norm, multiple studies have now demonstrated that this is not always true. Various mRNAs can target miRNA *∗*, rather than the guide strand, suggesting that even the passenger chain plays an important regulatory role in some biological processes [17, 18].

Changes in miRNA expression profiles were observed at various stages of NAFLD, including simple fatty liver (SFL), NASH, and liver fibrosis to HCC. In the SFL/NASH phase, expression of miR-122, miR-34a, and miR-192 was found to be significantly elevated in both human patient and animal models [19]. At the same time, the expression of miR-15b, miR125b, and miR-103 was observed to be increased in human patients [20–22], and the expression of miR-146b was decreased [23]. And in the progression and late stage of the disease, that is, the liver fibrosis/HCC stage, the expression of miR-122, miR-34a, and miR-16 was found to be elevated [24–26]. In one study, normal or HFD mice either received a 16-week running plan or remained sedentary. Studies have found that exercise attenuates hepatic steatosis in HFD mice, while miR-212 is overexpressed in the HFD liver and decreases after exercise [27]. This indicates that the expression of miRNA changes in the restored NAFLD, which is closely related to the process of NAFLD.

miRNA expression profiles changes have been described in human and animal NAFLD/NASH liver models [14]. Specifically, miRNA is an important regulator of liver pathophysiology and therefore influences NAFLD progression. Indeed, the role of specific miRNAs in NAFLD suggests that they can act as a potential diagnostic tool and biochemical marker. Below, we provide a summary of the main research on prominent miRNA.

### 2.1. miR-122

The most abundant (70%) miRNA expressed in the liver is miR-122. Transient inhibition of miR-122 expression in mice increased FA oxidation in the liver while decreasing serum cholesterol, as well as downregulating expression of cholesterol synthesis and FA synthase genes. In addition, miR-122 inhibition protects high-fat-fed mice from hepatic steatosis [28, 29]. Similarly, inhibiting miR-122 in nonhuman primates resulted in lowered cholesterol serum [30]. The above studies found that transient inhibition of miR-122 can improve liver lipid accumulation. However, germ cell knockout or liver-specific knockout miR-122 mice have lower than normal serum cholesterol, accompanied by hepatic triglyceride accumulation in juveniles. This relationship is mainly due to upregulation of miR-122 target genes associated with triglyceride biosynthesis and storage, such as Agpat1 (lysophosphatidic acid acyltransferase, alpha) and Cidec (DFFA-like effector c that induces cell death). In terms of diseases, miR-122 knockout mice are susceptible to NASH, fibrosis, hepatocellular carcinoma [31], time-dependent steatohepatitis, and HCC; restoration of miR-122a expression reduces disease symptoms and tumorigenesis [32]. The miR-122 knockout mouse model and the transient miR-122 inhibition animal model yield inconsistent results regarding miR-122 function. More studies are necessary to clarify such a contradiction. In HepG2 cells, inhibiting miR-122 increased expression of genes involved in lipid metabolism in the liver, whereas upregulating miR-122 significantly decreased gene expression of major lipogenic enzymes [33]. Further links with lipid synthesis include the downregulation of miR-122 in free FA-induced fatty liver cells, as well as in the streptozotocin and high-fat diet- (STZ-HFD-) induced NASH mouse model. A major mechanism of action for miR-122 is directly targeting YY1 mRNA in the downstream (YYH-SHP) signaling cascade to regulate lipid homeostasis [34]. The above studies indicate that miR-122 is involved in the regulation of hepatic lipid metabolism in NAFLD.

In addition, circulating miR-122 is significantly upregulated in the serum of NAFLD patients in a manner strongly correlated with disease severity [35]. Recently, individuals with fatty liver were found to exhibit upregulated circulating miR-122-5p [36]. Together, existing studies demonstrate that miR-122 expression differs between hepatocytes and blood (downregulated in the former, upregulated in the latter). Thus, we suggest that circulating miR122 levels are a useful biomarker that can improve the accuracy of noninvasive NAFLD diagnosis.

### 2.2. miR-34a

In the liver of HFD mice, metabolic syndrome patients, and NASH patients, miR-34a is the most upregulated miRNA [33]. The upregulation of miR-34a affects postprandial response and contributes to metabolic syndrome through targeting the coreceptor beta-Klotho, which attenuates liver fibroblast growth factor 19 signaling [37]. In addition, miR-34a can target SIRT1 to trigger FA *β*-oxidation while reducing FA synthesis and ectopic accumulation [38]. Liver miR-34a also influences nicotinamide phosphoribosyltransferase (NAMPT), the rate-limiting enzyme in NAD+ biosynthesis, thereby reducing NAD(+) and SIRT1 levels [39]. In turn, acetylation of SIRT1 target transcriptional regulators (peroxisome proliferator-activated receptor gamma coactivator 1-alpha (PGC-1*α*), sterol regulatory element-binding protein-1c (SREBP-1c), farnesoid X receptor (FXR), and nuclear factor-*κ*b (NF-*κ*B)) increases, causing steatosis, inflammation, and impaired glucose tolerance.

In NAFLD and NASH patients, miR-34a overexpression inhibits SIRT1 and dephosphorylates AMP kinase, thereby lowering the phosphorylation of HMG CoA reductase and altering liver cholesterol accumulation [40]. These results indicate that miR-34a-dependent disorders to cholesterol metabolism are likely involved in NAFLD progression. In vivo and in vitro experiments revealed that miR-34a was significantly upregulated in hepatocytes from fatty liver and liver tissues of HFD-fed mice [41]. Furthermore, miR-34a regulates lipid-metabolism gene expression, specifically targeting liver peroxisome proliferation-activator receptor *α* (PPAR*α*) and inhibiting very low-density lipoprotein secretion, while promoting hepatic steatosis and hypolipidemic effects in a HNF4-dependent manner [42]. Recent studies have found that miR-34a concentrations decrease after transduction of the forkhead family transcription factor class O 3 (FoxO3) in PA-induced biliary cells, suggesting that FoxO3 is a transcriptional regulator of miR-34a [43]. Furthermore, increasing miR-34a accelerates PA-induced lipid apoptosis in cholangiocarcinoma cells. In summary, miR-34a is important to the regulation of hepatic lipid metabolism and promotion of lipid apoptosis.

Unsurprisingly, given these connections, liver disease severity is associated with activation of the miR-34a/SIRT1/p53 pathway [44], and circulating miR-34a levels is associated specifically with NAFLD severity. Liver enzyme concentrations, as well as degree of fibrosis and inflammation, are all linked to miR-34a levels, explaining why miR-34 is positively correlated with simple steatosis progression and NASH severity [25]. In conclusion, the high correlation of miR-34a with specific NAFLD pathological features [45] makes the miRNA a good candidate for a noninvasive, diagnostic biomarker of the disease.

### 2.3. miR-33a/b

The dual miRNAs miR-33a/b are located in introns of the sterol regulatory element-binding protein-2 (SREBP-2) and SREBP-1 genes, respectively. Because SREBP-2 and SREBP-1 are involved in cholesterol and free FA metabolism, respectively [46], miR-33a/b play key roles in bile acid synthesis, FA oxidation, and cholesterol homeostasis [47, 48]. When miR-33 is inhibited in vivo, ATP-binding cassette transporter increases in the liver, promoting cholesterol reverse-transport pathways and elevating circulating high-density lipoprotein levels [17, 49]. These data indicate that miR-33 is important to lipid metabolism.

While their functions are related, the two miRNAs exert different physiological effects. Elevated cholesterol inhibits miR-33a concentrations, thus increasing the expression of cholesterol 7alpha-hydroxylase (CYP7A1) and cholesterol efflux transporters. In contrast, SREBP2 and miR-33a increase intrahepatic cholesterol through downregulating cholesterol efflux transporters and bile acid synthesis [50]. Additionally, miR-33 regulates the expression of FA-related genes [51, 52]. Specifically, miR-33a/b overexpression in vivo and in vitro reduces FA oxidation to cause triglyceride accumulation, whereas miR-33a/b targets SIRT6 [52], an important regulator of glucose metabolism. Both genes also target the AMP-dependent kinase *α*1 subunit (AMPK*α*1) [17, 52], which promotes *β*-oxidation, glucose uptake of fatty acids, and ATP synthesis. Upregulation of miR-33 also inhibits the insulin signaling pathway by targeting insulin receptor substrate-2 (IRS-2) [51, 52]. miR-33 plays a key role in lipid metabolism and insulin signaling pathways, suggesting that it could be involved in NAFLD development.

In addition to the effects of short-term anti-miR-33 treatment, a recent study found that long-term deletion of miR-33 increases triglyceride (TG) circulating levels and liver lipid deposition in HFD mice [53]. This finding underlines the need for extensive studies to observe long-term inhibition of miR-33 in nonhuman primates. Recently, it was found that liver miR33b*∗* expression was increased in obese people and was higher in nonalcoholic steatohepatitis (NASH) within obese people [54]. This indicates that miR33b*∗* liver expression is associated with NASH. For all this, the exact role of miR-33a/b in NAFLD and its feasibility as a new therapeutic strategy still require further investigation.

### 2.4. miR-21

The expression of miR-21 decreased in the liver of HFD-fed mice and Hepa1-6 cells treated with stearate. Furthermore, miR-21 upregulation increased the expression of FA binding protein 7 (FABP7) [55]. Serum miR-21 levels in NAFLD patients were lower than in healthy controls [56], while there was an increase in the expression of 3-hydroxy-3-methylglutaryl-co-enzyme A reductase (HMGCR). Subsequent in vitro experiments then confirmed that HMGCR is a target gene of miR-21. In apparent contradiction to these findings, however, miR-21 has also been observed to increase in both obese patients and HFD mice. A potential mechanism of action involves excessive unsaturated FA, which upregulates miR-21 expression. In turn, miR-21 downregulates phosphatase and tensin homolog (PTEN) expression, leading to steatosis [57]. In mice treated with an obesogenic diet, miR-21 deficiency alters the expression of multiple metabolism genes in such a way that insulin resistance, glucose tolerance, and hepatic steatosis are all aggravated [58].

The role of miR-21 in NASH has also been confirmed. Inhibiting miRNA-21 restores PPAR*α* expression in the NASH disease model, decreasing pathological symptoms in NASH-affected liver [59]. Liver, muscle, and serum biopsies of NAFLD patients revealed a major presence of miR-21/PPAR*α* axis [60]. The regulatory role of miR-21 in both NAFLD and HCC appears to be through its action in the HBP1-p53-SREBP1c pathway [61].

### 2.5. miR-155

Widely explored in tumors, miR-155 has recently been implicated in lipogenesis that could lead to NAFLD. Specifically, miR-155 levels in the liver and peripheral blood of NAFLD patients were significantly lower than in healthy controls [62]. Increased miR-155 expression can reduce lipid metabolism-related genes SREBP1 and fatty acid synthase (FAS), thus lowering intracellular lipid accumulation. Downregulating miR-155 expression has the opposite effect. In vivo studies have revealed that the reduction of hepatic lipid accumulation under miR-155 upregulation occurs through inhibiting Liver X receptor (LXR) *α*-dependent adipogenic signaling pathways. Fat synthesis signals and transforming growth factor (TGF) in white and brown adipose tissues can inhibit miR-155 expression to upregulation target genes and increase adipose tissue [63]. Therefore, miR-155 deficiency/downregulation should elevate white adipose tissue mass, potentially leading to obesity and NAFLD.

Several studies have also begun to investigate the role of miR-155 in inflammation. Steatosis was reduced in MCD-induced miR-155-deficient mice, but this change did not attenuate associated manifestations of hepatic inflammation [64]. However, in miR-155 (-/-) mice fed with HFD for 6 months, hepatic steatosis increased [65]. Therefore, we recommend more research on miR-155 pathophysiological mechanisms in lipid metabolism and inflammation of NAFLD and NASH patients.

### 2.6. miR-181

Already known for its involvement in various cancers, miR-181a is overexpressed in cirrhosis and HCC [66]. In hepatocytes of insulin resistance models and in serum of diabetic patients, miR-181a is elevated, suggesting that it plays a role in NAFLD, given that insulin resistance is a major symptom. Overexpressing miR-181a has multiple effects on insulin pathways. First, it reduces SIRT1 expression in hepatocytes to induce insulin resistance [67]. Given the link between fat and insulin resistance, it is unsurprising to find that NAFLD patients, as well as HFD-treated cows and ob/ob mice, all experienced significant elevation of miR-181a in blood and liver. Second, it inhibits the SIRT1-PGC-1*α* pathway and decreases insulin sensitivity, while increasing gluconeogenesis and lipid synthesis in dairy hepatocytes and HepG2 cells [68]. In addition, data from NAFLD patients and HFD-fed mice both showed that miR181b targets SIRT1 to regulate hepatic steatosis, a finding that was also confirmed in vitro [69]. Together, the available research suggests that liver miR-181 regulates insulin sensitivity and fat metabolism of NAFLD through SIRT1 inhibition.

## 3. LncRNAs

Long noncoding RNAs are either cis- or transacting in their capacity as transcriptional regulators [70, 71], interacting via enhancer RNAs (eRNAs) with transcription factors in the nucleus and cytoplasm [72]. Additionally, lncRNA is involved in posttranscriptional mRNA processing (splicing) [73, 74] and the sequestering of miRNAs away from their targets [75, 76]. Below, we summarize recent in vivo, in vitro, and clinical trials involving lncRNAs.

### 3.1. Maternally Expressed Gene 3 (MEG3)

Maternal expressed gene 3 (MEG3) encodes an lncRNA associated with several cancers. In CCl4-induced liver fibrosis models and human fibrotic livers, MEC3 expression was significantly reduced. Likewise, MEG3 expression was downregulated in human hepatic stellate cell line-LX-2 cells stimulated by transforming growth factor-*β*1 (TGF-*β*1) [77]. Overexpressing MEG3 in LX-2 cells appears to activate p53 and mediate cytochrome c release, thus promoting caspase-3-dependent apoptosis and inhibiting TGF-*β*1-induced cell proliferation. In human patients experiencing liver fibrosis and cirrhosis, liver MEG3 levels increased significantly [78]. The RNA-binding protein polypyrimidine bundle-binding protein 1 (PTBP1) interacts with MEG3, binding to the coding region of SHP, a key inhibitor of bile acid biosynthesis. This action results in rapid degradation of SHP mRNA and cholestasis of liver damage. The importance of MEG3 in the progression of liver fibrosis makes the gene a potential treatment target.

### 3.2. Alu-Mediated p21 Transcriptional Regulator (APTR)

Targeted silencing RNA screening successfully identified APTR, a novel lncRNA [79] that is involved in regulating cell cycle progression and cell proliferation. Studies in two animal models of liver fibrosis (CCl4 and bile duct ligation mice) have observed elevated APTR, a pattern also noted in liver fibrosis patients. Knocking down APTR can counteract *α*-SMA upregulation in TGF-*β*1-induced HSC in vitro, thus inhibiting HSC activation. Furthermore, APTR silencing attenuates liver fibrosis in CCl4-treated mice [80]. In the mouse liver fibrosis model, APTR accelerates cell cycle and promotes HSC proliferation through negatively regulating p21. In cirrhosis patients, serum APTR levels are higher than control, a difference also observed between patients with decompensated liver cirrhosis and those with compensated cirrhosis. Together, these data suggest that serum APTR may also be a valuable biomarker of liver fibrosis severity.

### 3.3. Metastasis-Associated Lung Adenocarcinoma Transcript 1 (MALAT1)

Metastasis-associated lung adenocarcinoma transcript 1 (MALAT1) plays a key role in tumor cell proliferation, migration, and invasion. After CCl4 treatment, fibrotic liver tissue and activated hepatic stellate cells (HSC) both exhibited MALAT1 upregulation. Knocking down MALAT1 alleviates liver fibrosis in mice through reducing Acta2 and Col1a1 levels. In addition, MALAT1 regulates RAS-associated C3 botulinum substrate 1 (Rac1) expression via the action of miR-101b as a competitive endogenous RNA (ceRNA), affecting primary HSC proliferation, cell cycle, and activation [81].

The role of MALAT1 in hepatic steatosis and insulin resistance has also been described. For instance, MALAT1 expression increased in the livers of ob/ob mice exposed to palmitic acid HepG2 cells and type 2 diabetes mellitus animal models. Moreover, MALAT1 can promote hepatic steatosis and insulin resistance via increasing nuclear SREBP-1c stability [82]. Additionally, MALAT1 regulates C-X-C motif chemokine ligand 5 expression in HepG2 cells and activates hepatic stellate cells (LX-2). Hyperglycemia and insulin both influence MALAT1 expression in HepG2 cells, whereas only insulin affects MALAT expression in LX-2 cells [83]. These various regulatory pathways imply that MALAT1 has multiple various roles in NAFLD development.

### 3.4. Plasmacytoma Variant Translocation 1 (PVT1)

Plasmacytoma variant translocation 1 (PVT1) is a novel lncRNA that is upregulated in various human cancers. In fibrotic liver tissue and activated HSC, PVT1 expression is upregulated [84]. In primary HSC, PVT1 knockdown inhibits HSC activation, resulting in HSC proliferation while reducing *α*-SMA and type I collagen. In vivo experiments it was also found that Pvt1 knockdown in CCl4-treated mice decreased liver collagen expression and reduced liver fibrosis. Moreover, PVT1 can function as a ceRNA for miR-152. Specifically, PVT1 competitively binds to miR-152 and inhibits PTCH1 expression through methylation, thereby activating the hedgehog pathway and promoting EMT in cirrhosis [84].

### 3.5. Steroid Receptor RNA Activator (SRA)

When SRA is knocked out in mice, the animals experience improved glucose tolerance and reduced hepatic steatosis, thus becoming resistant to HFD-induced obesity [85]. Knocking out SRA induces expression of hepatic fatty triglyceride lipase (ATGL) under normal diets and HFD. Moreover, liver SRA and ATGL expression are inversely regulated under fasting conditions. This study found that SRA inhibits ATGL promoter activity and regulates ATGL expression primarily by inhibiting the ability of FoxO1 to promote ATGL transcription. Thus, SRA may be involved in lipid metabolism under NAFLD.

### 3.6. Homeobox (HOX) Transcript Antisense RNA (HOTAIR)

When TGF-*β*1 activated hepatic stellate cells (HSC) in the CCl4-induced mouse liver fibrosis model and in human fibrotic liver, HOTAIR expression decreased significantly [86]. In LX-2 cells, HOTAIR upregulation increased levels of alpha-smooth muscle actin (*α*-SMA), alpha1(1) collagen (Col1a1), and fibrosis-associated genes, promoting liver fibrosis and cell proliferation. Further studies revealed that HOTAIR can function as a ceRNA for miR-148b, which promotes liver fibrosis via miR-148b regulation of DNA methyltransferase 1 (DNMT1)/MEG3/p53 pathway. Therefore, HOTAIR may be involved in the process of liver fibrosis, and of course it is necessary to further explore the role of HOTAIR in the progression of liver fibrosis in NAFLD.

### 3.7. Nuclear Enriched Abundant Transcript 1 (NEAT1)

NEAT1 is an lncRNA that is key regulator in tumors. Expression of NEAT1 is upregulated in HCC and influences HCC cell proliferation, invasion, and migration [87]. Additionally, NEAT1, acetyl-CoA carboxylase (ACC), and fatty acid synthase (FAS) mRNA are all significantly enhanced in both in vivo and in vitro models of NAFLD [88]. Knockdown of NEAT1 reduced FCA-induced elevation of ACC and FAS in hepatocytes while significantly attenuating NAFLD performance in SD rats. Further studies found that downregulating NEAT1 levels had a therapeutic effect on NAFLD rats through the mTOR/S6K1 signaling pathway [88]. These studies all showed that NEAT1 is involved in lipid metabolism of hepatocytes.

In addition, a significant increase in NEAT1 expression was found in activated mouse hepatic stellate cells (HSC) and carbon tetrachloride- (CCl4-) induced mouse liver fibrosis models. Knockdown of NEAT1 inhibits liver fibrosis in vivo and in vitro. Overexpression of NEAT1 promotes HSC activation through increasing *α*-SMA and Col1a1 [89]. The study found that the miR-122-KLF6 axis mediates the effect of NEAT1 on HSC activation. In addition, liver samples from cirrhosis patients exhibited reduced miR-122 levels, while NEAT1 and Kruppel-like factor 6 (KLF6) increased. This outcome indicates that the NEAT1-miR-122-KLF6 axis plays a role in liver fibrosis of mice and humans. Overall, existing studies suggest that NEAT1 may play a role in NAFLD, specifically in hepatic steatosis and fibrosis.

### 3.8. Other lncRNAs in the Akt/SREBP-1c Pathway

Recent studies have identified a new lncRNA, lncRNA suppressor of hepatic gluconeogenesis and lipogenesis (lncSHGL). In the liver of obese mice and NAFLD patients, researchers observed decreased expression of mouse lncSHGL and its human homologous lncRNA B4GALT1-AS1 [90]. Elevating liver lncSHGL can improve hyperglycemia, insulin resistance, and steatosis in obese diabetic mice. Mechanistic studies have found that lncSHGL recruits heterogeneous nuclear ribonucleoprotein A1 (hnRNPA1), activates the phosphatidylinositol 3-kinase (PI3K)/Akt pathway, and inhibits the mTOR / SREBP-1C pathway, thus ameliorating hyperglycemia and steatosis in obese mice.

Increased lncARSR, FA synthesis, and oxidation-related gene expression were observed in NAFLD patients, as well as in vitro and in vivo NAFLD models [91]. Knockdown of lncARSR improved liver lipid accumulation in vivo and in vitro. Importantly, lncARSR regulates SREBP-1c expression via the PI3K/Akt pathway, which in turn regulates hepatic steatosis. These studies revealed a novel role for lncRNA in regulating hepatic steatosis via the Akt/SREBP-1c pathway.

## 4. CircRNAs

CircRNA has many conserved binding sites for miRNAs and acts as a “miRNA sponge,” a term referring to competitive endogenous RNA (ceRNA) that interacts with miRNA AGO proteins to inhibit miRNA activity. The presence of miRNA sponges tends to increase miRNA target gene expression [92, 93]. This is a very important feature of circRNA functions, which can effectively bind and inhibit miRNA transcription, further affect the expression of downstream mRNA, form a circRNA-miRNA-mRNA pathway, and participate in various diseases [94]. Below, we summarized the role of circRNAs in NAFLD progression, focusing on the various circRNAs involved in disease development via the circRNA-miRNA-mRNA axis.

### 4.1. circRNA/miR-34a/PPAR*α*

Both circRNA_0046367 and circRNA_0046366 are endogenous regulators of miR-34a [95, 96]. The two circRNAs block miRNA/mRNA interactions with miRNA response element (MRE) and can abolish the latter's inhibitory effect on PPAR*α*. When the level of PPAR*α* is increased, its target gene carnitine palmitoyltransferase 2 (CPT2) and the acyl-CoA binding domain containing 3 (ACBD3) or the solute carrier family 27A (SLC27A) are activated, and the steatosis is reduced. These findings suggest that abnormal regulation of the circRNA_0046366 or circRNA_0046366/miR-34a/PPAR*α* signaling pathway may be a novel epigenetic mechanism leading to hepatic steatosis. This insight can provide a new direction for NAFLD treatment. Based on circRNA-miRNA-mRNA network analysis, decreased circRNA_021412 levels, and miR-1972 inhibition of LPIN1, the circRNA_021412/miR-1972/LPIN1 signaling cascade appears to be partially involved in regulating hepatic steatosis [97].

## 5. Conclusions and Future Perspectives

Nonalcoholic fatty liver disease is among the most prevalent chronic liver diseases globally. Its pathological features include lipid metabolism disorders, inflammation, and fibrosis. Thus, there is an urgent need for a better understanding of pathogenesis and accurate diagnostic strategies, particularly using noninvasive biomarkers. In this review, we summarized the emerging research on ncRNAs, a group of molecules that is heavily involved in NAFLD pathogenesis. ncRNA typically exhibits high cellular or tissue specificity, making them great candidates for predicting disease progression. As we learn more about the role of ncRNAs in NAFLD and other diseases, this class of molecules may become appropriate biomarkers and may also be an important reference for assessing disease severity. Among them, miRNAs are the most studied and have been implicated in lipid metabolism, insulin resistance, inflammation, fibrosis, as well as HCC development. Given their correlation with disease severity, miRNAs could be biomarkers used in early noninvasive diagnosis and assessment of NAFLD severity. Serum miRNA levels may be used as sensitive biomarkers for early detection of NAFLD. Studies have shown that the serum level of miR-122 in mice with a methionine-choline deficiency (MCD) diet has increased 40-fold, far exceeding serum alanine aminotransferase (ALT) (4.8-fold) and aspartate aminotransferase (AST) (3.3-fold) [98]. It was also found that elevated levels of serum miR-122 were observed in NAFLD rats even without elevated ALT [99]. This indicates that miR-122 is more sensitive than cytokeratin (CK)-18, ALT, or AST in detecting NASH and predicting liver fibrosis in patients with NAFLD [20]. Recently, panels containing several serum miRNAs, or a combination of miRNAs and other biochemical indicators, have been shown to have a high diagnostic value for NAFLD and a higher predicted NASH potential than other biomarkers [100, 101]. Despite this strong potential, the clinical application of miRNAs remains in its infancy. Miravirsen is a locked nucleic acid-modified DNA phosphorothioate antisense oligonucleotide that inhibits miR-122 activity and sequesters it in a stable heteroduplex. This is the first injectable miRNA-targeted drug for the treatment of HCV that has entered clinical trials, with effective and long-term reduction in viremia in treated HCV-infected patients without serious side effects [102, 103]. So we have reason to hypothesize that Miravirsen may has an effect on the treatment of NAFLD. Treatment of mice and African green monkeys with antagomiRs against miR-33a / b was found to be effective in increasing liver ABCA1 expression, resulting in increased high density lipoprotein (HDL) and decreased plasma levels of very high density lipoprotein (VLDL) triglycerides [17]. Therefore, oligonucleotides mimicking endogenous downregulated miRNAs in NAFLD may also be used therapeutically [104]. However, there are still many problems to be solved in the study of non-coding RNA. Contradictory results from different studies must still be resolved, and currently, miRNA detection methods have not yet been screened. Furthermore, comparative research analyzing how serum and plasma miRNAs may differ is lacking. Besides these methodological issues, further research is also necessary before we can fully address a number of major biological questions pertaining to ncRNA and NAFLD. For instance, we currently do not have a good grasp on lncRNA and circRNA function and mechanism of action in steatosis, inflammation, and fibrosis during NAFLD. We also need to investigate the relationship between lncRNA/circRNA and miRNA interactions, as well as their roles as ceRNA and miRNA sponges.
